# New Biscoumarin Derivatives: Synthesis, Crystal Structure, Theoretical Study and Antibacterial Activity against *Staphylococcus aureus*

**DOI:** 10.3390/molecules191219868

**Published:** 2014-11-28

**Authors:** Di Qu, Jing Li, Xiao-Hui Yang, Zi-Dan Zhang, Xiao-Xing Luo, Ming-Kai Li, Xia Li

**Affiliations:** 1 Department of Pharmacology, School of Pharmacy, the Fourth Military Medical University, Xi’an 710032, China; E-Mails: qudi3157@126.com (D.Q.); xxluo3@fmmu.edu.cn (X.-X.L.); 2 School of Chemistry and Chemical Engineering, Xi’an University, Xi’an 710065, China; E-Mails: lijing_518@126.com (J.L.); yangxh1127@aliyun.com (X.-H.Y.); 3 Department of Physics, School of Science, Tianjin University, Tianjin 300072, China; E-Mail: zhangzidan@tju.edu.cn; 4 Department of Neurosurgery, Xijing Hospital, the Fourth Military Medical University, Xi’an 710032, China

**Keywords:** biscoumarin, *Staphylococcus aureus*, minimum inhibitory concentration

## Abstract

Five novel biscoumarins **1**–**5** were synthesized and characterized. In these compounds, two classical asymmetrical intramolecular O–H···O hydrogen bonds were used to stabilize the whole structures and the HB energies were performed with the density functional theory (DFT) [B3LYP/6-31G*] method. The five compounds were evaluated for their* in vitro* antibacterial activities against *Staphylococcus aureus* (*S. aureus* ATCC 29213), methicillin-resistant *S. aureus* (MRSA XJ 75302), vancomycin-intermediate *S. aureus* (Mu50 ATCC 700699), and USA 300 (Los Angeles County clone, LAC) by the means of minimum inhibitory concentration and time-kill curves.

## 1. Introduction

*Staphylococcus aureus* (*S. aureus*) is a major pathogen, and the leading cause of healthcare-associated infections [1]. It can cause severe sepsis complicated by acute renal failure and respiratory failure requiring intensive care [2,3]. However, many strains of which are now resistant to almost all the antibiotics. Naturally occurring strains of methicillin-resistant *Staphylococcus aureus* (MRSA) were first reported in England in 1961 [4], not long after the introduction of semisynthetic penicillins. The prevalence rates of MRSA in hospitals in some Asian countries, such as Taiwan, China, Japan, and South Korea, range from 70% to 80% [5,6]. Because the efficacy of novel therapeutic agents against MRSA stays largely unexplored, the treatment failure of MRSA infections makes this field be of great interest in the future.

4-Hydroxycoumarin derivatives have attracted much interest in several fields and represent an important class of organic heterocycles that can be found in many natural or synthetic drugs [7,8,9]. These compounds possess versatile biological activities, such as anticoagulant, insecticidal, antihelminthic, hypnotic, and antifungal activities, phytoalexin production, and HIV protease inhibition [10,11,12,13]. Biscoumarins consisting of a 4-hydroxycoumarin dimer have received considerable attention because of their special molecular structures (two intramolecular O–H···O hydrogen bonds) and diverse biological properties through chemical modifications (different substituents on the central linker methylene). Recognizing the considerable importance of the compounds, the researchers focused on the synthesis of biscoumarin derivatives.

**Figure 1:**
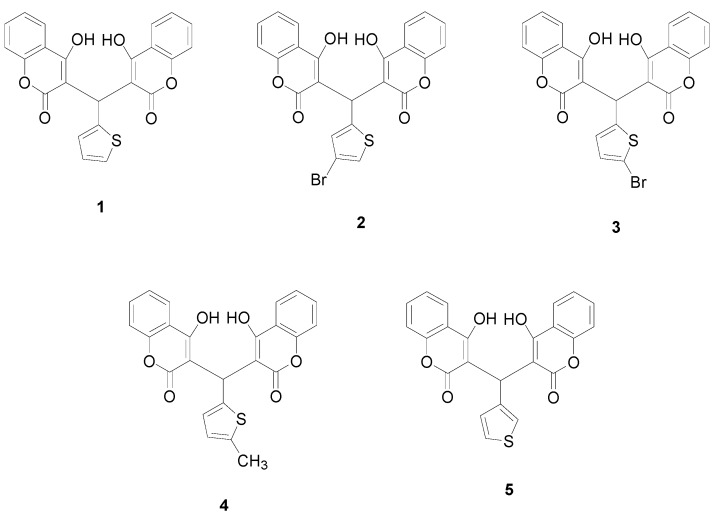
Chemical structures of compounds **1**–**5**.

In the current study, a series of biscoumarins (Figure 1), namely, 3,3'-(2-thienyl-methylene)-bis-(4-hydroxycoumarin) (**1**), 3,3'-(4-bromo-2-thienyl-methylene)-bis-(4-hydroxycoumarin) (**2**), 3,3'-(5-bromo-2-thienylmethylene)-bis-(4-hydroxycoumarin) (**3**), 3,3'-(5**-**methyl-2-thienylmethylene)-bis-(4-hydroxycoumarin) (**4**) and 3,3'-(3-thienyl-methylene)-bis-(4-hydroxycoumarin) (**5**), were synthesized and their antibacterial activities were investigated in detail. A possible relationship between such hydrogen-bonded structures and their antibacterial activities was further studied by theoretical calculations.

## 2. Results and Discussion

### 2.1. Molecular Structure

**Figure 2:**
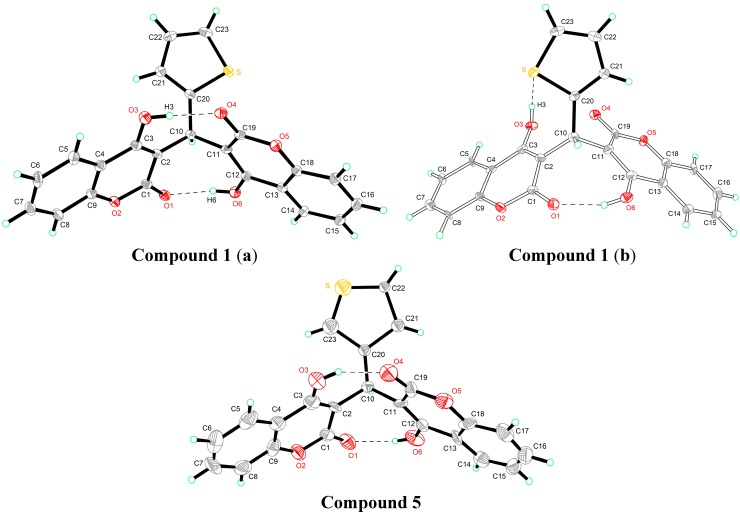
Crystal structures of compounds **1** and **5**.

The crystal structures of compounds **1** and **5** are given in Figure 2. In the crystal structure of compound **1**, two crystallographically independent molecules are present in the asymmetric unit. The whole molecule is disordered over two orientations with refined site occupancies of 0.75:0.25, and two 4-hydroxycoumarin fragments are linked by a methylene bridge, wherein one hydrogen atom is replaced with a 2-thienyl residue. However, the two components differ with respect to the reversed twist directions of two 4-hydroxycoumarin molecules. In the major component (a), two classical intramolecular hydrogen bonds were found; each links a coumarin hydroxyl and carbonyl group [*d*(O_3_–O_4_) = 2.592 Å, *d*(O_1_–O_6_) = 2.697 Å]. In the minor component (b), one classical intramolecular hydrogen bond is between a hydroxyl group of one coumarin fragment and a lactone carbonyl group of another coumarin fragment; the other classical intramolecular hydrogen bond is between a hydroxyl group of one coumarin fragment and thiophene ring S atom.

Like compound **1**, in the crystal structure of compound **5**, two 4-hydroxycoumarin moieties are linked through a methylene bridge, wherein one hydrogen atom has been replaced with a 3-thienyl residue. In addition, two classical asymmetrical intramolecular O–H···O hydrogen bonds [*d*(O_3_–O_4_) = 2.587 Å, *d*(O_1_–O_6_) = 2.709 Å] between two 4-hydroxycoumarin fragments were used to stabilize the whole structure.

### 2.2. Quantum Chemical Calculations

#### 2.2.1. Geometric Parameters of Compounds **1**–**5**

**Figure 3:**
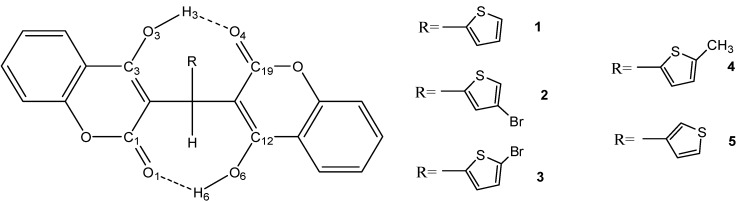
Schematic representation of compounds **1**–**5**.

The fully optimized molecular structures of compounds **1**–**5** with atomic numbering calculated at B3LYP level of theory are shown in Figure 3. Selected calculated geometric parameters under three different basis sets (6-31G*, 6-31+G**, and 6-311G*) and experimental geometric parameters of compounds **1** and **5** are presented in Table 1.

**Table 1:** Experimental and calculated parameters of the selected bond lengths and bond angles of compounds **1** and **5**.

Name Definition	Compound 1				Compound 5			
	X-ray	6-31G*	6-31+G**	6-311G*	X-ray	6-31G*	6-31+G**	6-311G*
O_3_···O_4_	2.592	2.639	2.614	2.643	2.587	2.643	2.621	2.648
O_1_···O_6_	2.697	2.704	2.695	2.711	2.709	2.698	2.686	2.706
C_19_=O_4_	1.234	1.229	1.232	1.222	1.192	1.229	1.232	1.222
C_1_=O_1_	1.235	1.233	1.236	1.226	1.214	1.233	1.236	1.226
C_1_-O_2_	1.361	1.372	1.370	1.371	1.309	1.373	1.371	1.372
C_9_-O_2_	1.355	1.367	1.369	1.366	1.324	1.368	1.369	1.366
C_19_-O_5_	1.352	1.376	1.373	1.375	1.318	1.377	1.375	1.377
C_18_-O_5_	1.354	1.367	1.368	1.365	1.389	1.367	1.368	1.365
C_10_-C_20_	1.514	1.521	1.522	1.520	1.412	1.529	1.529	1.528
C_1_-C_2_-C_10_	113.63	114.14	114.12	113.94	113.38	114.62	114.65	114.40
C_3_-C_2_-C_10_	126.92	126.33	126.55	126.52	126.59	125.76	125.91	125.96
C_10_-C_11_-C_19_	119.17	118.92	119.07	118.71	115.55	119.12	119.37	118.90
C_10_-C_11_-C_12_	122.30	121.42	121.47	121.58	127.08	121.40	121.37	121.56
C_2_-C_10_-C_11_	112.56	113.41	113.58	113.51	110.78	112.96	113.15	113.03
C_2_-C_10_-C_20_	116.66	114.66	114.89	114.82	114.91	114.48	114.43	114.59
C_11_-C_10_-C_20_	111.34	113.97	114.02	113.95	115.93	115.02	115.38	115.01
C_3_-C_2_-C_10_-C_11_	84.78	83.91	83.52	84.56	88.12	87.60	87.46	88.20
C_2_-C_10_-C_11_-C_19_	82.36	83.36	83.13	83.08	87.43	81.07	80.59	80.82
C_1_-C_2_-C_10_-C_20_	136.87	134.30	133.69	134.30	134.62	135.07	134.21	135.02
C_19_-C_11_-C_10_-C_20_	50.79	50.27	51.08	50.88	45.85	52.90	53.89	53.37

The values under three different basis sets are very close, and the calculated results agree with the experimental findings. The average discrepancy of the selected bond lengths and bond angles between theoretical and experimental data is less than ±0.02 Å and ±2°, respectively. B3LYP/6-31G* exhibited sufficient agreement with experimental data and lower computational cost, so further theoretical study was performed at this level.

#### 2.2.2. Estimation of the Single and Total HB Energies in Compounds **1**–**5**

To obtain single and total HB energies of the five compounds, structure optimization was performed to elucidate stable PES structures. We take compound **5** as an example to estimate single and total HB energies. Compound **5** which was stabilized by two HBs is the global minimum structure (**5ab**); however, there also could be two higher energy structures (**5a** and **5b**) stabilized by one HB respectively.

The O_6_—H_6_···O_1_ HB energy was estimated from the energy difference between **5ab** and **5a**, $E(O_{6}—H_{6}\cdot \cdot \cdot O_{1})=E_{\text{5ab}}^{\text{coor}}-E_{\text{5a}}^{\text{coor}}$, calculated to be −50.56713 kJ/mol (Table 2). **5a** is a global minimum structure with one HB (O_3_—H_3_···O_4_). The O_3_—H_3_···O_4_ HB energy was estimated from the energy difference between **5ab** and **5b**, $E(O_{3}—H_{3}\cdot \cdot \cdot O_{4})=E_{\text{5ab}}^{\text{coor}}-E_{\text{5b}}^{\text{coor}}$, calculated to be −65.4615915 kJ/mol (Table 2). **5b** was obtained from the global minimum structure of **5ab**, but H_3_ was rotated around the C_3_—O_3_ bond until O_3_—H_3_···O_4_ HB rupture occurred. From the above HB energies values, we can see that O_3_—H_3_···O_4_ HB strength is stronger than that of O_6_—H_6_···O_1_, which is consistent with the fact that the distance of O_3_—O_4_ (2.587 Å) is shorter than that of O_6_—O_1_ (2.709 Å). The total HB energy in compound **5**, calculated by the equation $2E_{\text{5ab}}^{\text{coor}}-(E_{\text{5a}}^{\text{coor}}+E_{\text{5b}}^{\text{coor}})$, was estimated to be −116.0287215 kJ/mol (Table 2). Like compound **5**, the O_3_—H_3_···O_4_ HB energy for compounds **1**–**4** is also stronger than O_6_—H_6_···O_1_ HB energy. In addition, the total HB energies of compounds **1**–**4** are −125.4805215, −122.274786, −123.529775, −126.743387 kJ·mol^−1^, respectively (Table 2).

**Table 2:** Total electronic energies (in Hartree) and HB energies (in kJ/mol) of hydrogen bonded conformers of compounds **1**–**5** calculated at B3LYP/6-31G* level of theory.

System	Total Electronic Energies ^a^	E(O_6_—H_6_···O_1_)	E(O_3_—H_3_···O_4_)	E(Total HB)
**1ab**	−1734.116678			−125.4805215
**1a**	−1734.096324	−53.439427		
**1b**	−1734.089239		−72.0410945	
**2ab**	−4305.229694			−122.274786
**2a**	−4305.209554	−52.87757		
**2b**	−4305.203262		−69.397216	
**3ab**	−4305.225972			−123.529775
**3a**	−4305.206014	−52.399729		
**3b**	−4305.19888		−71.130046	
**4ab**	−1773.408037			−126.743387
**4a**	−1773.387526	−53.8516305		
**4b**	−1773.380274		−72.8917565	
**5ab**	−1734.116974			−116.0287215
**5a**	−1734.097714	−50.56713		
**5b**	−1734.092041		−65.4615915	

^a^: ZP corrected.

### 2.3. Minimal Inhibitory Concentration (MIC) Assay

Four *S. aureus* bacterial strains, including one drug-sensitive *S. aureus* (*S. aureus* ATCC 29213) strain and three MRSA strains (MRSA XJ 75302, Mu50, USA 300 LAC), were used in the systematic analysis of the antibacterial activities of compounds **1**–**5*** in vitro*. As shown in Table 3, among the compounds, compound **1** exerted the most potent bactericidal effects against nearly all types of *S. aureus* tested, and its MIC values ranged from 8 to 32 μg/mL. By contrast, the other compounds exerted weaker bactericidal effects against *S. aureus*, and their MIC values exceed 32 μg/mL for *S. aureus* (ATCC 29213) and the three MRSA strains. Compared with the MIC values of the above compounds, the MIC values of ceftazidime, ceftriaxone, gentamicin and piperacillin against *S. aureus* (ATCC 29213) strains were lower (less than 8 μg/mL) but were higher against resistant strains at varying degrees.

**Table 3:** MIC of compounds **1**–**5** and antibiotics in Mueller-Hinton Broth Culture.

Drug	MIC (ug/mL)			
	*S. aureas* (ATCC 29213)	MRSA (XJ 75302)	Mu50 (ATCC 700699)	LAC (USA 300)
Compound **1**	8	8	16	32
Compound **2**	64	64	64	128
Compound **3**	64	64	64	128
Compound **4**	32	32	32	32
Compound **5**	>256	>256	>256	>256
Ceftazidime	8 (S)	>256 (R)	256 (R)	64 (R)
Ceftriaxone	2 (S)	>256 (R)	256 (R)	32 (R)
Gentamicin	0.12 (S)	64 (R)	32 (R)	0.25 (S)
Piperacillin	2 (S)	>128 (R)	>128 (R)	64 (R)

S means drug susceptibility; R means drug resistance. Ceftazidime, ceftriaxone, gentamicin, and piperacillin as control antibiotics exert anti-bacterial effects on the drug-susceptible *S. aureas* strain (ATCC 29213). MRSA (XJ 75302) and Mu50 (ATCC 700699) are resistant to all of the control antibiotics, whereas LAC (USA 300) is susceptible to gentamicin but resistant to the other control antibiotics.

### 2.4. Bacterial Growth Inhibition

**Figure 4:**
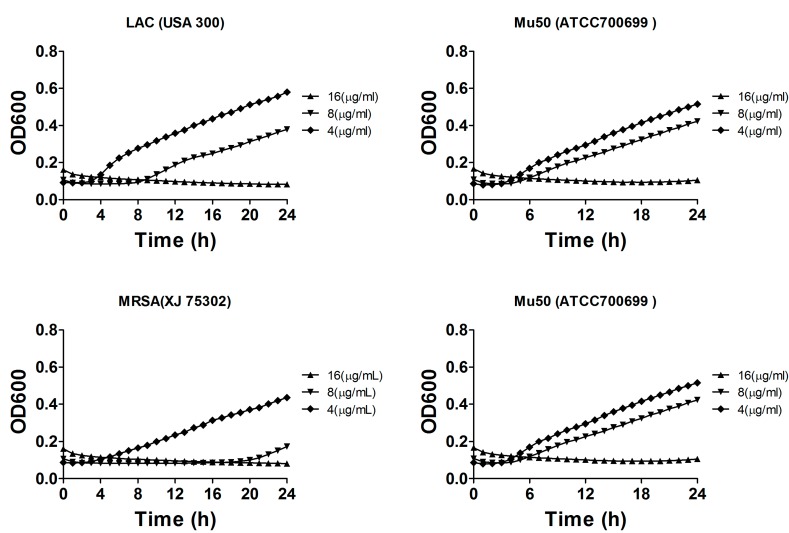
Concentration-dependent inhibition of compound **1** on the growth of *S. aureus* ATCC 29213, MRSA XJ 75302, Mu50, and MRSA USA 300 LAC. The cells were cultured in liquid culture medium and treated with different concentrations of compound **1**.

We further investigated the growth inhibitory and bactericidal effects to gain insight into the mode of action of compound **1**. Additional experiments were performed to determine the growth rate of *S. aureus* in liquid medium containing the different concentrations of the compound. Compound **1** was added to cultures at concentrations of 4, 8 or 16 μg/mL to evaluate the growth inhibitory effects on *S. aureus* ATCC 29213, MRSA XJ 75302, Mu50, and MRSA USA 300 LAC. As shown in Figure 4, compound **1** inhibited the growth of these pathogens and exhibited almost completely growth inhibition on these pathogens at 8 or 16 μg/mL. Similar to the results of the MIC values, the other compounds hardly showed any inhibitory effects on these pathogens at these concentrations (data was not shown). *S. aureus* growth in MH broth without any compounds, which was used as the control sample, did not exhibit any significant growth inhibitory effect. The analysis of bacterial growth inhibition showed that aside from exerting antibacterial activities on *S. aureus,* compound **1** also inhibited the growth of the drug-sensitive and drug-resistant *S. aureus* strains.

## 3. Experimental Section

### 3.1. Apparatus and Materials

IR spectra (400–4000 cm^−1^) were obtained using a Bruker Equinox-55 spectrophotometer. ^1^H-NMR spectra were obtained (at 400 MHz) using a Varian Inova-400 spectrometer. Mass spectra were obtained using a micrOTOF-Q II mass spectrometer. The melting points were taken on a XT-4 micro melting apparatus, and the thermometer was uncorrected. All antibiotics used were purchased from the National Institute for the Control of Pharmaceutical and Biological Products (Beijing, China). All other chemicals and solvents were of analytical grade. MRSA (XJ 75302) was isolated from cultures of sputum samples from patients in Xijing Hospital (Xi’an, China). *S. aureus* strain (ATCC 29213) was purchased from the Chinese National Center for Surveillance of Antimicrobial Resistance. Mu50 (ATCC 700699) and USA 300 (LAC) were purchased from MicroBiologics (Saint Cloud, MN, USA).

### 3.2. Synthesis and Characterization of Compounds **1**–**5**

Compounds **1**–**5** were synthesized according to the methods of a previous report [14]. A mixture of thiophene-2-carbaldehyde (or 4-bromo-thiophene-2-carbaldehyde, 5-bromo-thiophene-2-carbaldehyde, 5-methyl-thiophene-2-carbaldehyde and thiophene-3-carbaldehyde) (10 mmol) and 4-hydroxycoumarin (20 mmol) was dissolved in EtOH (100 mL). A few drops of piperidine were added, and the mixture was stirred for 3 h at room temperature. After reaction completion as determined by TLC, water was added until precipitation occurred. After filtering the precipitates, they were sequentially washed with ice-cooled water and ethanol and then dried under vacuum.

*3,3'-(2-Thienyl-methylene)-bis-(4-hydroxycoumarin)* (**1**): m.p. 206–207 °C. IR (KBr pellet cm^−1^): 1665, 1612, 1567, 1514, 1356, 767 cm^−1^. ^1^H-NMR (CDCl_3_, δ, ppm): 11.803 (s, 1H), 11.290 (s, 1H), 8.017–8.073 (m, 2H), 7.617–7.656 (q, 2H), 7.399–7.420 (d, 4H), 7.214–7.227 (d, 1H), 6.940–6.962 (t, 1H), 6.855–6.864 (t, 1H), 6.200 (s, 1H). HRMS (ESI^+^): *m*/*z*: calcd. for C_23_H_14_SO_6_: 441.0403 [M+Na^+^]; found: 441.0411.

*3,3′-(4-Bromo-2-thienyl-methylene)-bis-(4-hydroxycoumarin)* (**2**): m.p. 207–208 °C. IR (KBr pellet cm^−1^): 1666, 1620, 1571, 1533, 1363, 1246, 1143, 911, 760 cm^−1^. ^1^H-NMR (CDCl_3_, δ, ppm): 11.871 (s, 1H), 11.307 (s, 1H), 8.045–8.090 (t, 2H), 7.651–7.690 (t, 2H), 7.422–7.443 (d, 4H), 7.144 (s, 1H), 6.789 (s, 1H), 6.169 (s, 1H). HRMS (ESI^+^): *m*/*z*: calcd. for C_23_H_13_BrO_6_S: 518.9508 [M+Na^+^]; found: 518.9532.

*3,3′-(5-Bromo-2-thienyl-methylene)-bis-(4-hydroxycoumarin)* (**3**): m.p. 217–218 °C. IR (KBr pellet cm^−1^): 1673, 1620, 1552, 1492, 1356, 1318, 1205, 1099, 971, 767 cm^−1^. ^1^H-NMR (CDCl_3_, δ, ppm): 11.890 (s, 1H), 11.299 (s, 1H), 8.036–8.090 (q, 2H), 7.646–7.685 (t, 2H), 7.420–7.441 (d, 4H), 6.910–6.919 (d, 1H), 6.634–6.647 (q, 1H), 6.118–6.122 (d, 1H). HRMS (ESI^+^): *m*/*z*: calcd. for C_23_H_13_BrO_6_S: 518.9508 [M+Na^+^]; found: 518.9589.

*3,3′-(5**-**Methyl-2-thienyl-methylene)-bis-(4-hydroxycoumarin)* (**4**): m.p. 209–210 °C. IR (KBr pellet cm^−1^): 1673, 1605, 1567, 1492, 1363, 1311, 1265, 1212, 1107, 767 cm^−1^. ^1^H-NMR (CDCl_3_, δ, ppm): 11.828 (s, 1H), 11.279 (s, 1H), 8.062 (s, 2H), 7.628–7.667 (t, 2H), 7.389–7.432 (t, 4H), 6.608–6.644 (d, 2H), 6.160 (s, 1H), 2.438 (s, 3H). HRMS (ESI^+^): *m*/*z*: calcd. for C_24_H_16_SO_6_: 455.0560 [M+Na^+^]; found: 455.0599.

*3,3′-(3-Thienyl-methylene)-bis-(4-hydroxycoumarin)* (**5**): m.p. 226–227 °C. IR (KBr pellet cm^−1^): 1661, 1600, 1539, 1521, 1349, 1240, 1090, 910, 760 cm^−1^. ^1^H-NMR (CDCl_3_, δ, ppm): 11.584 (s, 1H), 11.308 (s, 1H), 8.023–8.093 (q, 2H), 7.631–7.670 (q, 2H), 7.418–7.436 (d, 4H), 7.306–7.325 (q, 1H), 7.037 (s, 1H), 6.890–6.902 (d, 1H), 5.986 (s, 1H). HRMS (ESI^+^): *m*/*z*: calcd. for C_23_H_14_SO_6_: 441.0403 [M+Na^+^]; found: 441.0465.

### 3.3. X-ray Crystallography

For X-ray diffraction experiments, single crystals of compounds **1** and **5** were both grown from methanol. The X-ray diffraction data were collected on a Bruker SMART APEX II CCD diffractometer equipped with a graphite monochromated Mo Kα radiation (λ = 0.71073 Å) by using the ω-2θ scan technique at room temperature. The structure was solved by direct methods using SHELXS-97 [15] and refined using the full-matrix least squares method on *F*^2^ with anisotropic thermal parameters for all non-hydrogen atoms by using SHELXL-97. Hydrogen atoms were generated geometrically. The crystal data and details concerning data collection and structure refinement are given in Table 4. Molecular illustrations were prepared using the XP package. Parameters in CIF format are available as Electronic Supplementary Publication from Cambridge Crystallographic Data Centre.

**Table 4:** Crystal data, data collection and structure refinement.

Parameter	Compound 1	Compound 5
Formula	C_23_H_14_O_6_S	C_23_H_14_O_6_S
*M*r	418.40	418.40
Temperature/K	113(2)	293(2)
Crystal system	Monoclinic	Monoclinic
Space group	*P*2_1_/*n*	*P*2_1_/*n*
*a*/Å	7.7250(8)	7.7983(4)
*b*/Å	8.9250(10)	8.9785(5)
*c*/Å	26.537(2)	26.6473(19)
α/°	90	90
β/°	96.319(9)	96.514(6)
γ/°	90	90
*V*/Å^3^	1818.5(3)	1853.72(19)
*Z*	4	4
*D*_calc_/g·cm^−3^	1.528	3.009
μ(Mo Kα)/mm^−1^	1.951	1.711
θ range/°	3.35 to 72.59	2.40 to 25.00
Reflections collected	15733	6656
No. unique data[*R*(int)]	3541[0.0619]	3261[0.0307]
No. data with *I* ≥ 2σ(*I*)	3242	1892
*R*_1_	0.0603	0.2049
ω*R*_2_(all data)	0.1362	0.6273
CCDC	889260	1014707

### 3.4. Quantum Chemical Calculations

All calculations were carried out using the Gaussian 09 package [16]. Density functional theory (DFT) [17,18], Becke’s three-parameter hybrid function (B3LYP) [19], and LYP correlation function [20,21] were used to fully optimize all the geometries on the energy surface without constraints. To obtain precise results that are in conjunction with experimental results, three basis sets, namely 6-31G*, 6-31+G**, and 6-311G*, were tested. Frequency calculations at the B3LYP (with basis sets 6-31G*) level of theory were carried out to confirm stationary points as minima and to obtain the zero-point energies and the thermal correlation data at 1 atm and 298 K.

### 3.5. Minimal Inhibitory Concentration (MIC) Assay

Based on the CLSI broth microdilution method [22], the determination of minimum inhibitory concentrations (MICs) via microdilution assay was performed in sterilized 96-well polypropylene microtiter plates (Sigma–Aldrich, St. Louis, MO, USA) in a final volume of 200 μL. Bacteria were grown overnight in nutrient broth. Mueller–Hinton (MH) broth (100 μL) containing bacteria (5 × 10^5^ CFU/mL) was added to 100 μL of the culture medium containing the test compound (0.12 μg/mL to 256 μg/mL in serial twofold dilutions). The plates were incubated at 37 °C for 20 h in an incubator. About 50 µL of 0.2% triphenyl tetrazolium chloride (TTC), a colorimetric indicator, was added to each well of microtiter plates and incubated at 35 °C for 1.5 h. The TTC-based MIC was determined as the lowest concentration of oxacillin that showed no red color change indicating complete growth inhibition.

### 3.6. Bacterial Growth Inhibition

To obtain the time–kill curves for methicillin-susceptible *S. aureus* and MRSA, the synthetic compounds and antibiotics were added to strain cultures to a final concentration of 4, 8 or 16 μg/mL [23]. The strains were cultivated in the automated Bioscreen C system (Lab Systems, Helsinki, Finland) by using an MH broth culture medium. The working volume in the wells of the Bioscreen plate was 300 µL, which comprised 150 µL of the MH broth and 150 µL of the drug solution. The temperature was controlled at 35 °C, and the optical density of the cell suspensions was measured automatically at 600 nm in regular intervals of 10 min for 20 h. Before each measurement, the culture wells were automatically shaken for 60 s. Statistical data for each experiment were obtained from at least two independent assays performed in duplicate.

## 4. Conclusions

The emergence of vancomycin-resistant *S. aureus* and treatment failure of MRSA infections urgently requires developing new antimicrobials [24,25,26]. In the current work, we report the synthesis, crystal structures, and antibacterial of the novel biscoumarin derivatives, and observed their activity on clinical isolates strains including methicillin-susceptible or methicillin-resistant *S. aureus*. Both MICs and bacterial growth inhibition results showed that compound **1** exerted potent bactericidal effects against almost all *S. aureus* tested including the MRSA.

Two intramolecular O—H···O HBs in the five compounds were considered as an important factor for biological activity by assisting the molecule to attain the correct configuration. The calculated results are creditable because of the fully optimized molecular structures of compounds **1** and **5** calculated at B3LYP level of the theory using three different basis sets (6-31G*, 6-31+G** and 6-311G*) were in agreement with their available X-ray data.

The total HB stabilization energies in compounds **1**–**4** were estimated to be −125.4805215, −122.274786, −123.529775 and −126.743387 kJ/mol, which is higher than that of compound **5** (−116.0287215 kJ/mol). These values suggest that the most potent antibacterial activity of compound **1** is basically consistent with the stronger HB strengths. Additional experiments should be carried out to further define the mechanism underlying its anti-bacterial activity and evaluate the correlations of its drug efficacy* in vivo*.
